# The Demon of Socrates
*"Du Démon de Socrate," par L. F. Lélut, Membre de l'Institut. Paris, 1856.


**Published:** 1857-07-01

**Authors:** 


### Art. II.—THE DEMON OF SOCRATES.*

The glory was departing from Athens. Above a thousand years had elapsed since its foundation; it had passed through all gradations, from a condition of barbarism, when its heroes were little better than skin-clad freebooters, to one of refinement, which made it the centre of the civilized world. It had most powerfully influenced the destinies of Greece, by successfully opposing almost single-handed the entire power of Xerxes; and its military renown had culminated in the immortal victories of Marathon, Salamis, and Platæa. The pride, the arrogance which manifested themselves after these great events, stirred up against it the other States of Greece, and determined them to its destruction. But far worse than external enemies were those that arose within. Enervating luxury and brutal intemperance gradually invaded all ranks of society, and a general demoralization was the result. Then ensued that certain sign of decaying power, or of a State shaken to its very foundations, that prelude to its fall—rapid changes of forms of government, from rabid democracy to oligarchy and despotism.

Yet, menaced as she was both from within and without, Athens was still, and long continued to be, the favoured seat of learning and the arts. In the period to which we allude (about the fifth century B.C.), she numbered amongst her celebrated sons such intellectual giants as Pericles, Phidias, Eschylus, Sophocles, Euripides, Aristophanes, Socrates, Xenophon, and Plato. It is curious and interesting to analyze the elements of civilization in times which could produce great intellects like these. Personally, a superficial refinement of manner barely professed to conceal a gross licentious immorality, assuming forms which forbid even a faint allusion in these times—publicly, the most shameless undisguised venality characterised their courts, misnamed, of justice. In their external relations, the wars, undertaken on the slightest pretexts, were wars of extermination—the cities were destroyed, and the inhabitants killed or enslaved. Occasionally even yet, the favour of the gods was propitiated by human sacrifices. On the morning of the battle of Platæa, Aristides sent to Themistocles three nephews of Xerxes, whom he had taken prisoners; and, by the advice of an augur, they were sacrificed to Bacchus, to purchase his favour. Thus, although the Greeks were no longer anthropophagi, their gods were.† The thousands of deities that were admitted, and in some sort worshipped, were but the coarsest embodiments of human pas-

* "Du Démon de Socrate," par L. F. Lélut, Membre de l'Institut. Paris, 1856.

† Lélut.

sions—drunken, gluttonous, indecent creations, half despised and half feared by their votaries—somewhat more powerful than men, but susceptible of being duped by them; and equally with them subject to an unalterable, irrevocable fatality. But, as a contrast or background to the portrait of Socrates, and a sketch of his teaching, nothing can be more effective than a statement of the condition of what was called philosophy before his day. Now that exact science has made at least some progress in the world—now that facts are in some measure recognised as necessary elements in theorizing—now that observation, at least so far as science is concerned, is allowed to be a necessary preliminary to dogmatism—it is scarcely possible for the mind to realize or credit the futile nature of the questions which occupied the acutest minds; or the arbitrary, wordy, windy, unreasoning manner in which they were settled by one school, or unsettled by another. Now that the majority of men recognise a material and an immaterial nature, it is difficult to picture the chaotic ideas held on the subject of the universe, its origin, its nature, its laws.

"All the philosophers," says Mr. Grote,* "of the fifth century B.C., prior to Socrates, inheriting from their earliest poetical predecessors the vast and unmeasured problems which had once been solved by the supposition of divine or superhuman agents, contemplated the world physical and moral, all in a mass; and applied their minds to find some hypothesis which would give them an explanation of this totality, or at least appease curiosity by something which *looked like* an explanation. What were the elements out of which sensible things were made? What was the initial cause or principle of those changes which appeared to our senses? What was change? was it generation of something integrally new, and destruction of something pre-existent—or was it a decomposition and re-combination of elements still continuing?"

Others were occupied in demonstrating the impossibility of change or motion. Parmenides denied that change of either colour or form could take place. Zeno† showed by logic that motion was impossible, a proposition supported strongly by Melissus and many others; they upheld likewise the unity of matter, that the real ultra-phenomenal substance was One, unchangeable and undivisible; whilst their opponents maintained that it was not One but Many, divisible, moveable, and changeable. These, and other equally urgent matters, occupied the minds of all thinking men. Observation and induction seem to have been unknown or practically ignored; with the exception of some few discoveries in astronomy and mathematics, science was in complete infancy; physical science was represented only by such theorists as Thales, Leucippus, Democritus, and Empe-

* "Hist. of Greece," vol. viii.
† Not Zeno the Epicurean, but Zeno of Elea.

docles, reasoning vaguely upon air, water, fire, atoms, and their combinations, by means of Friendship or Enmity, as causes of motion or change. A complete bar also to progress in observation was the opinion so generally held, that the senses were delusive, and not to be trusted in any matter. Gorgias professes to demonstrate "that nothing exists; that if anything exist, it is unknowable ; and granting it even to exist, and to be knowable by any one man, he could never communicate it to others." (Grote, p. 503.) It may be questioned whether some of the ontological doctrines of our own times are much more explanatory. Cicero, in his *Academic Questions*, gives a brief summary of the cosmogonic systems of this age, fully illustrating the entire ignorance of natural science which prevailed, and the tendency to rest in forms of words.

"Is (Thales) enim infinitatem naturæ dixit esse, ex qua omnia gignerentur. Post ejus auditor Anaximenes, infinitum äera, sed ea, quæ ex eo orientur, definita ; gigni autem terram, aquam, ignem, tum ex his omnia. Anaxagoras materiam infinitam, sed eas particulas similes inter se, minutas ; eas primum confusas, postea in ordinem adductas mente divina. Xenophanes, unum esse omnia, neque id esse mutabile. Parmenides, ignem, qui moveat terram, quæ ab eo formatur, Leucippus, plenum et inane.—Pythagorei ex numeris et mathematicorum initiis proficisci volunt omnia."

Content thus to remain bound up in forms of words without meaning, debarred from further progress by the legitimate way of observation by distrust of the senses, because these revealed to them phenomena which would not be thus formulated, philosophers degenerated into mere sceptics, doubting nature, doubting themselves, doubting their gods. "Respecting the gods," says Protagoras, "I neither know whether they exist, nor what are their attributes; the uncertainty of the subject, the shortness of human life, and many other causes, debar me from this knowledge." Philosophy, it was evident, must receive some new impulse, be diverted into new channels, or it must perish. This impulse was not long wanting. In the workshop of Sophroniscus, a sculptor, was a youth, who was destined to introduce a new philosophy, new morals, new manners, and almost a new religion ; and all this without any formal teaching, without professing any code of opinions, without forming any school. His one weapon, with which he warred against the vices, the scepticisms, the obstinacy, and the self-conceit of the world and the sophists, was the great negative arm of Grecian analysis, the cross-examining Elenchus. It may almost be said to have been created or invented by Socrates (although Zeno* seemed to be in some

* Of Elea.

measure acquainted with its value); it may truly be said to have perished with him.

"Where are we to look for a parallel to Socrates, either in or out of the Grecian world? The cross-examining Elenchus, which he not only first struck out, but wielded with such matchless effect and to such noble purposes, has been mute ever since his last conversation in the prison; for even his great successor Plato was a writer and lecturer, not a colloquial dialectician. No man has ever been found strong enough to bend his bow, much less sure enough to use it as he did. His life remains as the only evidence—but a very satisfactory evidence—how much can be done by this sort of intelligent interrogation; how powerful is the interest which it can be made to inspire; how energetic the stimulus which it can apply in awakening dormant reason, and generating new mental power."*

Simple, unostentatious, and temperate, amid the luxuries and temptations of the most luxurious city in the world—pure among the most impure—virtuous amongst the most venal—clear-sighted to see through the sophisms and verbiage which overlaid and swamped all thought—he devoted all the energies of his hardy nature, all the tendencies of a long life, to the practice and inculcation of virtue. St. Augustine says of him, that he was the first who, leaving celestial matters as too obscure or abstruse to be penetrated by man, reduced philosophy to the reformation ot manners; and Cicero says,† *Socrates autem primus philosophiam devocavit è cœlo, et in urbibus collocavit, et in domos etiam introduxit, et coegit de vitâ, et moribus, rebusque bonis, et malis quærere.* Forsaking as either unworthy or impossible of solution the questions which hitherto had been supposed to *constitute* philosophy; as to the One or the Many—motion, divisibility or stability of matter, change or permanency, &c.; he continually turned his investigations and the thoughts of his interlocutors to *human* affairs. What is good? What is beautiful? What is just or unjust? What are temperance, courage, cowardice? What is a city, and what a citizen? What is piety? Such were the questions with which he was ever occupied, leading his fellow-citizens to the comprehension of the great truths involved in them; whilst in his own person he afforded a bright and consistent example of all the virtues which he taught. Stern rebuker of vice—uncompromising enemy to injustice, even in high places—living reproach to impurity—terrible enemy to the darkening of counsel by words without knowledge,—he was found too far, morally and intellectually, in advance of his countrymen to be tolerated by them, and they put him to death. But it remained for the wisdom of the nineteenth century to make the great and

* Grote's "Hist. of Greece," vol. viii., p. 664.

† "Tusc. Quæst.," lib. v.

somewhat startling discovery that Socrates was A MADMAN! That we may not be liable to the imputation of misrepresentation, we quote literally from M. Lélut's recent work the following passage :—

"Reste une troisième et dernière opinion . . . . et cette opinion, qui consiste à dire que Socrate était un théosophe, un visionnaire, et pour dire le mot, UN FOU—cette opinion est la seule vraie."

This opinion is founded upon the contested point of the demon or familiar spirit of Socrates; M. Lélut considering it as an hallucination of hearing, and perhaps of sight also; and thus arriving at the conclusion that Socrates was of unsound mind. A brief sketch of his life and character is necessary as a preliminary to the examination of this point.

Socrates was born about the year 469 B.C. His father was Sophroniscus, the sculptor, and his mother Phanarete, a midwife. Of his childhood little or nothing is known, except that his father was advised by an oracle to leave the child to his own natural instincts, as he had within himself a guide worth a thousand teachers. Notwithstanding this, he was brought up to his father's profession, for which he had little vocation; and, according to Diogenes Laertius, might often have been observed, chisel in hand, lost in thought, arrested in his uncongenial but necessary toil by some vein of philosophic inquiry. He made some progress in the art of sculpture; and as late as the time of Pausanias, a group of his workmanship was to be seen at the entry of the citadel of Athens. From the necessity for manual labour he was at last released by the generosity of Crito, at what period of life does not appear.* At first he seems to have pursued the ordinary curriculum of study, including the physical sciences of that time, with geometry, music, and the art of oratory: he soon, however, concluded that these studies were either useless, or shrouded in impenetrable darkness; and thenceforth he devoted himself entirely to the study of morals, and of the duties of men and citizens.

"These efforts," says M. Lélut, "of renovating moral philosophy were not made from a professorial chair, nor in a place set apart for tuition, nor at set times, in the intervals of which he thought of other things. They were made in all places, at all times—in Athens, as with the army—in the street, as at the dining-table—in the workshops of artisans, as in the boudoir of Callisto or of Theodote."

In the street, the forum, the baths, the gymnasium—wherever the people, particularly the youth, were congregated, there was Socrates with his never-ending questions. Of the origin, reasons, and method of this system of interrogation, he himself gives an account in his Apology as related by Plato. It appears that a friend of his, named Chærepho, being at Delphi, ventured to

* Most probably when about nineteen years of age.

inquire of the oracle who was the wisest man, and received for answer that none was wiser than Socrates.

"I reasoned thus with myself: What does the god mean? what is the enigma? For I am not conscious that I am wise, either much or little. . . . . . Afterwards, with considerable difficulty, I had recourse to the following method of searching out his meaning."

He then describes how he went to one of the greatest politicians of the day and questioned him, and how he found that he was only wise in the opinions of others and in his own, but not really so.

"I thereupon endeavoured to show him that he fancied himself to be wise, but was not really so. Hence I became odious, both to him and many others who were present. When I left him, I reasoned thus with myself: I am wiser than this man, for neither of us appear to know anything great or good; but he fancies he knows something, although he knows nothing; whereas I, as I do not know anything, so I do not fancy I do. In this trifling particular, then, I appear to be wiser than he."

His researches amongst all classes of the learned led him to the same conclusion—he everywhere found that he was making himself odious by exposing ignorance and pretence; but feeling that to elucidate the meaning of the oracle was of paramount importance, he continued the same course of interrogation.

"At last, therefore, I went to the artizans. For I was conscious to myself that I knew scarcely anything, but I was sure that I should find them possessed of much beautiful knowledge. And in this I was not deceived; for they knew things which I did not, and in this respect they were wiser than I. But, O Athenians, even the best workmen appeared to me to have fallen into the same error as the poets; for each, because he excelled in the practice of his art, thought that he was very wise in other most important matters; and this mistake of theirs obscured the wisdom that they really possessed. I therefore asked myself, in behalf of the oracle, whether I should prefer to continue as I am, possessing none either of their wisdom or their ignorance, or to have both, as they have. I answered therefore to myself and to the oracle, that it was better for me to continue as I am."

His general conclusion is, that all being alike ignorant of any real wisdom, human knowledge being of little worth, he only can be wiser than his fellows who is aware of this ignorance.

"Still, therefore, I go about and search and inquire into these things, in obedience to the god, both among citizens and strangers, if I think any one of them is wise; and when he appears to me not to be so, I take the part of the god, and show him that he is not wise."

It is related that when Sir H. Davy was making his great researches into the constitution of the earths and alkalies, some of the chemical professors felt greatly aggrieved at having their previous notions disturbed. A noted professor at a Scotch university refused all recognition of these researches, as long as he decently could do so. When ultimately compelled to make some allusion to them, he did it very briefly, accompanying it with the opinion that Mr. Davy was "a very tiresome person." Such in an eminent degree must have been the judgment of many of the Athenians with reference to Socrates. All those who, under the pressure of his *Elenchus* were reduced to silence, palpable contradictions, or tacit confessions of ignorance, would be inclined to view him with little favour. Those who winced under his crushing irony—those whose vices he lashed so unsparingly—those whose secret souls he laid bare for their own inspection and appreciation—all would hate him much more than they would despise themselves. A notorious instance occurred in the person of Critias, who at one time was a constant follower of Socrates. Having spoken earnestly to Critias on the subject of one of the vices then fashionable, and he having paid no attention to his remonstrances, *λεγεται τον Σωκρατην, αλλων τε πολλων παροντων και του Ευθυδημου, ειπειν, οτι ϋικον (τι) αυτῳ δοκοιη ο Κριτιας, επιθυμων Ευθυδημου προσκνησθαι, ωσπερ τα ϋϊδια τοις λιθοις*. An eminently disagreeable person must Critias have thought Socrates; and he did not forget it.

The remarks made by our great English satirist upon Swift would have been very applicable to Critias:—"If undeterred by his great reputation you had met him like a man, he would have quailed before you, and not had the pluck to reply; and gone home, and years after written a foul epigram about you—watched for you in a sewer, and come to assail you with a coward's blow and a dirty bludgeon." For years afterwards, when he had long left the society of Socrates, and was one of the Thirty Tyrants, he remembered his sarcasm, and not knowing how to find matter of accusation against Socrates individually, so pure and blameless was his life, he inserted in the laws that "none should teach the art of disputation," and took every opportunity of using his power to annoy him.—Polus, a pert, loquacious young man, who had put himself forward to answer Socrates in the place of Gorgias the rhetorician, went away smarting under his irony, and doubtless thinking him very objectionable.

"*Socr.* Most excellent Polus! we get ourselves friends and sons for this express purpose, that when we, through being advanced in years, fall into error, you that are younger being with us may correct our life both in deeds and words. If, then, Gorgias and I have fallen into any error in our arguments, do you who are present correct us; you ought to do so. And I wish that if any of the things that have been granted appear to you to have been improperly granted, you would retract whatever you please; only I beg you beware of one thing.

"*Pol.* What is that?

"*Socr.* That you would restrain that prolixity of speech which at first you attempted to employ.

"*Pol.* What? shall I not be allowed to speak as much as I please?

"*Socr.* You would indeed be very badly treated, my excellent friend, if, having come to Athens, where of all Greece there is the greatest liberty of speech, you alone should here be deprived of this liberty. But set this against it; if you speak in a prolix manner, and will not answer a question put to you, shall I not be badly treated if I am not allowed to go away and not listen to you?"

But leaving for the present the method and matter of the teaching of Socrates, it is time to inquire into the grounds upon which M. Lélut considers it right to class him amongst madmen.

His persuasion of a special religious mission was one of the leading peculiarities in the character of Socrates. This is more than once alluded to in his defence before his judges. "This duty," he says, alluding to his mission to cross-examine his fellow-citizens upon points of virtue and piety, "has been enjoined me by the Deity, by oracles, by dreams, and by every mode by which any other divine decree has ever enjoined anything to man to do." And again:—

"Perhaps, however, it may appear absurd that I, going about, thus advise you in private, and make myself busy, but never venture to present myself in public before your assemblies, and give advice to the city. The cause of this is that which you have often and in many places heard me mention: because I am moved by *a certain divine and spiritual influence*, which also Melitus, through mocking, has set out in the indictment. This began with me from childhood, being a *kind of voice* which, when present, always diverts me from what I am about to do, but *never* urges me on. This it is which opposed my meddling in politics; and it appears to me to have opposed me very properly."

In this and passages of similar import are to be found the entire elements of this allegation. Socrates was constantly in the habit of expressing himself as moved and influenced by the god, ο θεος; by a divine or spiritual influence—το δαιμονιον—or το δαιμονιον σημειον—translated by some substantively as the DEMON, and the sign of the Demon; by a voice—φωνη—checking him, but never urging him on.

There are three modes of interpretation of these forms of expression—three hypotheses to account for the facts. The first is, that Socrates used these words to express, figuratively and forcibly, the motions of conscience. The second is, that it was a system of deceit practised by him to increase his power over the minds of his hearers, and propagated by his followers to add to the dignity of their master, as having been under immediate Divine guidance.

The third opinion is the one adopted or suggested by M. Lélut, that Socrates was subject to hallucinations of hearing—

perhaps also of sight; that he was therefore a visionary—a madman!

We will briefly trace the arguments and considerations relative to the psychological history of Socrates, by which M. Lélut endeavours to support this view. He introduces the subject thus:—

"Since Plato and Xenophon, all the writers who have examined with any precision the thoughts and acts of Socrates, have united, under the generic title of DEMON, or Familiar Spirit, all that part of those thoughts and acts relative to the singularities of his life, which is beyond the common course. I mean his inspirations, his presentiments, his prophecies, and especially that divine voice which he heard, or said that he heard; which impelled him to no action, but deterred him from many which might have been unjust or dangerous; a voice which enabled him at many times to give to his friends and disciples counsels, which they always found good to follow, and dangerous to neglect.

"In recognising and exalting the purity and sublimity of his life, the admirable consecutiveness of his thoughts and actions, all writers have remarked something extraordinary and eccentric in this life exclusively consecrated to the triumph of one or two ideas, and to the accomplishment of the same design. . . Not only was he a *singular* youth, but he had been a singular child—of a meditative spirit doubtless; of great capacity; but assuredly of an equally great peculiarity: of this no further proof is needed than the counsel of the Oracle to leave him to his own natural instincts, and his own confession that from a child he had felt the influence of the *genius* in question.

"Socrates, then, had from his earliest years a *singularity* (I lay stress upon the word) which his mature age was not to belie. Was he not in reality a singular man, this Socrates, clothed in the same mantle in all weathers and seasons—walking barefoot upon the ice as upon the parched and heated soil of Greece—dancing and leaping, often alone, by fits and starts—leading, in the eyes of the vulgar, the most eccentric life—having no other occupation than to pervade the public places and the workshops of the artisans—pursuing every one with his questions and his irony—receiving nothing from friends or disciples, yet asking them for a coat when necessary—acquiring, in fine, by his conduct and manners, such a reputation for eccentricity, that he was afterwards surnamed by Zeno the Epicurean, as Cicero relates, *Atticus scurra*, the buffoon of Athens—what we should now call an original?

"Notwithstanding these things, the Oracle of Delphi, when consulted by Chærepho as to who was the wisest man of Greece, replied—Sophocles is wise, Euripides is wiser, but Socrates is wisest of men. Thereupon Socrates, who wished to understand the meaning of this, commenced amongst all professions in Athens that singular course of interrogations, which by demonstrating the ignorance of those who were accounted wise, drew upon him the hatred of so many.

"Psychologically speaking, the matter might have rested there, and he have been only accounted a singular and extraordinary man, if he had not from his infancy been disposed to take the inspirations of his conscience for the voice of a supernatural agent. This thought, too lively, too ardent, too much disposed to *transfer itself to the exterior*, to clothe itself with personality, to become an image, or at least an audible voice, took in effect this last form ; and then commenced all at once the *hallucinations* of Socrates—that is to say, the most undeniable form of alienation (*l'espèce de folie la plus irréfragable*)."

M. Lélut considers the actual insanity of Socrates to have commenced at the siege of Potidæa, where he served with distinction as an oplite, and where he had a fit of abstraction, which appeared like an ecstasy or trance. We find an account of this given by Alcibiades in the "Banquet," which it may be well to give entire :—

"But what this patient man did do and dare during the campaign there, it is worth while to hear. For while he was thinking of some question for himself, he stood from the dawn investigating it ; and as he did not succeed, he did not desist, but stood still investigating it. It was mid-day, and some persons perceived him, and wondering said that Socrates had been standing from the morning thinking upon something. At length some Ionian soldiers, when it was evening, having supped—for it was then summer—brought out their ground-litters, and partly slept in the cold, and partly kept watch, whether he would stand there all night. And he did stand until the dawn appeared and the sun rose; after which he departed, having first offered a prayer to the sun."

In commenting upon this relation, M. Lélut observes that we must either deny the facts, or "recognise in them the commencement of a condition which no one would voluntarily experience, even to possess all the virtue and all the glory of the son of Sophroniscus." Not to interfere with the general course of the argument, we would merely suggest that this does not appear to us an exhaustive view of the subject, but that recognising the facts, we need not attach so serious an import to them. It is not impossible that he who had turned his back upon an old, worn-out, effete system of philosophy, and who out of the depths of his own thought had eliminated the great truths of the immortality of the soul, and the certainty of a future state of rewards and punishments,—who from a chaotic Polytheism had arrived at the belief in ONE GOD, the CREATOR and upholder of all things,—it is not impossible that such a man may have been so wrapt and lost in the opening immensity and profundity of these considerations, as to become insensible to surrounding objects for even so long a time as is here mentioned. Archimedes and Newton were not suspected of madness because of their frequent and prolonged reveries ; and their problems yield in vastness to those that engaged this colossal mind.

M. Lélut relates one or two other instances of his reveries, or, as he would style them, ecstasies; and then proceeds to quote from the "Dialogues of Plato" most of the passages where Socrates speaks of himself as influenced by the god (ο θεος), the demon (το δαιμονιον), or the voice (η φωνη). Some of them are certainly remarkable. In the "Philebus," Socrates uses this expression:—

"At the moment of passing the water, I felt the divine signal (το δαιμονιον σημειον), which is familiar to me, and the presence of which always arrests me at the moment of action. I seemed to hear a voice which forbid me to cross." This would, *so far*, appear to argue a belief in some personality; but an examination of the following remark modifies this impression much. "Such as you see me, I am a diviner (ειμι δη μαντις μεν)—not a very able one, truly; I resemble those whose writing is only legible to themselves—I know enough for my own purposes. *The human soul has a prophetic power.*" Here the same powers are spoken of as personal—not as communicated from without.

Some of the most remarkable passages, however, are those in which Socrates speaks of his influence over his pupils, in which some mysticism may readily be discovered by those engaged in the search after it. In the "Theages," Socrates relates a conversation of his own with Aristides, the son of Lysimachus, by way of illustrating this influence. He represents Aristides as saying:—

"I am going to relate a thing which might appear incredible, but which is nevertheless true. I have never learnt anything from you, as you very well know. And yet, when with you, even in the same house, though not in the same room, I have always profited in wisdom; when in the same room, I have advanced more rapidly still; but most of all when, being in the same room, I had my eyes fixed upon yours; or most especially if I sat near you and touched you."

Socrates then continues:—

"Such, dear Theages, is the commerce that one may have with me. *If it please the god* (τῳ θεῳ), you will, by being near me, profit much, and in little time; but *if not*, your efforts will be in vain. Consider then whether it will not be more advantageous to you to attach yourself to some master who will certainly be useful to you, rather than to follow one who cannot answer for anything."

M. Lélut remarks upon this:—

"I cannot refrain from pointing out how strange in their nature and development, how truly maniacal (*veritablement maniaque*) in principle, are the beliefs and pretensions announced in the last passage. Here is Socrates, who not only imagines that he receives divine influences and inspirations, and hears a divine voice; but who, by reason of this privilege, believes that he possesses a similar influence, even at a distance, upon his friends, his disciples, and even strangers; an influence independent of word or look, exerting itself even through walls. In truth, it is impossible to hear or see anything more extravagant or more characteristic of madness; et les hallucinés, qui, sous nos yeux, prétendent envoyer ou recevoir à distance des influences physiques, magnétiques, franc-maçonniques, ne s'expriment pas autrement que Socrate, et ne sont, sous ce rapport, *pas plus fous qu'il ne l'était.*"*

M. Lélut then passes on to comment upon the expressions used by Socrates in his defence, with reference to the divine influence under which he acted; and he is of opinion that these develop, in the most formal manner, as obvious and inveterate hallucinations of hearing as were ever observed by a physician. The passages are too long to cite textually. In the "Apology," Socrates repeatedly uses all the forms already quoted—professing in all matters to *act* under the immediate influence, guidance, and direction of the divinity (του θεου), which, be it remarked, is attended by no voice; but *to be restrained from action* by the *voice*, or Demon—the φωνη, or δαιμονιον σημειον. He tells the Athenians that he has pursued the course of life which they so reprobate, influenced by the god, through the medium of dreams, oracles, &c. He tells them that he has *refrained* from preparing a defence, because the Voice prevented him. Upon all this M. Lélut puts the same literal interpretation as before noticed.

In the determination to represent Socrates as the victim of hallucinations, he extends them from the ear to the eye, and insists that Socrates *saw* his Demon as well as heard it—though he himself emphatically disclaims such a vision, and moreover disputes its possibility. He says that there are gods, who preside over the well-being of men, but that only their works are visible in results; and that neither they themselves nor their immediate agents (as the thunderbolt) are visible or palpable at any time. ("Memorabilia," lib. iv.) Yet on the strength of a vague conjecture of Apuleius, M. Lélut says he has no doubt that the eye was subject to a corresponding hallucination with the ear; and an equally unsatisfactory testimony states that the sense of touch was similarly affected. In his general summary he says:—

"Socrates had ecstasies, almost accessions of catalepsy, as happened to him at the siege of Potidæus, and elsewhere. Soon these ecstasies assumed the character of more definite hallucinations, shorter, but more frequent; hallucinations of the general tact or sensibility internal or external; hallucinations especially of hearing, and most probably of vision. Nothing assuredly can be more extraordinary; but, at the

* In this and some other passages we prefer giving the original, for the obvious reason that a translation would scarcely be credited.

same time, nothing can be more irrefragable as a criterion of insanity than these hallucinations."

Socrates had undoubtedly some faith in dreams of a certain character—he spoke in mysterious phraseology also of the prophetic powers of the spirit of man. From all these considerations combined, M. Lélut concludes that *Socrates was insane.*

It is undoubtedly true that there are many hitherto "unrecognised forms of insanity," developing themselves in peculiarities and changes of temper, habits, general disposition, morals, and the like. But it appears to us to be a retrograde step, and one likely to throw discredit upon psychological inquiry, and to subvert all useful generalization, to look for marks of insanity in a close adhesion to the modes of belief of any particular age and country, a poetical or figurative mode of expression, and a habit of reverie—to see mental aberration in slight eccentricities of conduct, in defiance of the evidence of a long life characterized by the acutest and most comprehensive intelligence that perhaps ever adorned man; a purity and blamelessness of life and manners which not even his bitterest enemies could impeach; and a death such as might well have crowned, and added new lustre to, the life of the greatest of ancient philosophers. Such a verdict is only equalled by that passed by the same authority on the great Pascal, who is pronounced to have been hallucinated, and *thus* insane, on the strength of a parchment found after his death, sewed within his doublet, on which were written some rather unconnected mysticisms, which may *possibly* be interpreted to have reference to some supposed vision.*

When analysed, the evidence upon which Socrates is here pronounced insane may be considered under these heads:—(1) His belief in a special divine mission; (2) his frequent references to a spiritual monitor or Voice, called by some his Demon or Genius; (3) his reveries or ecstasies; (4) his belief in dreams; (5) his belief in, and claims of possessing, a prophetic power; and (6) certain eccentricities of habit and manner.

1. Socrates was in the constant habit of expressing himself as acting under the direct influence and impulse of the god. He was so far in advance of the great majority, if not all, of his countrymen, as to recognise one Supreme Power, who was not a practical nonentity in the world, but a Creator and an upholder, and who exercised a paternal care over his creatures. As a *stimulus to action* he always recognised this power, piously acknowledging that all ability and all disposition to action came from this source. When Aristodemus inquired into the nature of this influence, he advised him to pay special and assiduous court to the gods, that

* "L'Amulette de Pascal." Par F. Lélut. 1846.

they may exert a similar one over him: thus, in this instance, at least, disclaiming any peculiar theurgic manifestation.

2. The case is somewhat different with regard to the especial monitor or Voice, to which he so constantly alludes. Though acknowledging one Supreme Power, he did not entirely forsake the Polytheism of his country; but believed in certain inferior orders of spirits, called Demons, who were the immediate agents in carrying out the Supreme will. Of these he believed that one (or more) was appointed to every man to be his guardian,—to perform near him certain providential functions. In the "Phædo," giving his friends a summary of his creed, amongst other things he says, "that each person's demon, who *was assigned to him while living*, when he dies, conducts him to some place where they that are assembled together must receive sentence, and then proceed to Hades with that guide, who has been ordered to conduct them from hence thither. But then having received their deserts, and having remained the appointed time, another guide brings them back hither again, after many and long revolutions of time." This belief seems not to have been contrary to that of the ancient world generally, "insomuch," says Mr. Grote on this subject, "that the attempts to resolve phenomena into general laws were looked upon with a certain disapprobation, as indirectly setting it aside." This may be granted then, that he believed in the existence of demons with a special mission to act upon nature and man, one of which at least attended upon every man. But he frequently spoke of a something peculiar to himself, an influence, a voice, which diverted him from any act which he was about to commit, but never urged him on, or suggested anything. In this particular it differs essentially from the *motor* influence noticed under the former head. But this restraining power, which he said had always forbidden him to enter on public life, and prevented his preparing any formal defence at his trial—this power, although spoken of by many writers as his Demon or Genius—he himself *never personified*, but spoke of it as a "kind of voice," or a "certain divine and spiritual influence;" it was never more than το or τι δαιμονιον, with or without the word σημειον added—or φωνη, the Voice. Critically, it is acknowledged that the former phrase, which M. Lélut always translates "the Demon," is only properly to be understood adjectively, even when the substantive is not expressed; and therefore that it can but be translated "something spiritual." M. Cousin, the learned translator of the works of Plato, holds this view as undeniable; and one of the highest critical authorities in Europe, Schleiermacher, says—"*Semper adjectivè poni hanc vocem, neque in ullo Xenophontis aut Platonis aut aliorum Scriptorum æqualium loco substantive de deo accipi debere.*"

Cicero also interprets it as "*divinum quoddam.*" It seems to have been a highly figurative method of speaking of conscience and reason, which he conceived to be stronger in him than in other men (and in so far peculiar to him); inasmuch as his recognition of the Divine Power, and the reverence to be paid thereto, was more intense and constant. For it will be found that, almost invariably after speaking of being prevented by this "divine influence" from adopting any particular course, he gives some *human* and *rational* grounds for such a determination. Thus, in his "Apology," having related how this Voice had always prevented him meddling in public affairs, he adds :—

"For be well assured, O Athenians, if I had long since attempted to intermeddle with politics, I should have perished long ago, and should not have at all benefited either you or myself. And be not angry with me for speaking the truth; for it is not possible that any man should be safe who sincerely opposes you, or any other multitude, and who prevents many unjust and illegal actions from being committed in a city; but it is necessary that he who in earnest contends for justice, if he will be safe for but a short time, should live privately, and take no part in public affairs."

And when he stated that the Voice had prevented his preparing beforehand any defence, he adds *the reason why* :—

"For what has befallen me appears to be a blessing; and it is impossible that we think rightly who suppose that death is an evil. . . . To a good man nothing is evil, neither while living nor when dead; nor are his concerns neglected by the gods. And what has befallen me is not the effect of chance; but this is clear to me, that now to die, and be freed from my cares, is better for me."

It is unnecessary to multiply instances. There is scarcely an occasion when the Voice is not accounted for in a manner equally rational.

But it may be asked, what was the meaning of those strong expressions, which seemed to imply that there was an actual audible voice? An examination of a passage in the "*Crito*" will show that these were purely poetical or figurative. His friend Crito had come early one morning to the prison, after his condemnation, with the intent to persuade him to escape. Socrates takes the opportunity to discuss with Crito the duties of a citizen; and, in the course of the conversation, shows that he must obey the established laws, at whatever cost to himself. He shows that the city has nurtured him and protected him— that he has been most especially a voluntary citizen of Athens, never having left it, except in time of war; and so recognised the right and power which her laws possessed over him. He then personifies these laws, and supposes them to be addressing him, pointing out all the benefits he has received from his country, and all the evil that might result from his attempting to evade the decree, concluding thus:—

"'But now, Socrates, you depart (if you *do* depart) unjustly treated, not by *us*, but by men; but should you escape, having thus disgracefully returned injury for injury, and evil for evil; having violated your own compacts and conventions which you made with us, and having done evil to those to whom you least of all should have done it—namely, yourself, your friends, your country, and us—both we shall be indignant with you so long as you live; and our brothers, the laws in Hades, will not receive you favourably, knowing that you attempted, as far as you were able, to destroy us. Let not Crito, then, persuade you to do what he advises, rather than we.'

"*These things, my dear friend Crito, be assured I hear, as the votaries of Cybele seem to hear the flutes. And the sound of these words booms in my ear, and makes me incapable of hearing anything else.*"

And thus in language as strongly, if not more strongly implying an audible voice, than any which he uses with regard to the so-called Demon, he gives the summary of the argument which by his own reason he has just eliminated in conversation with Crito. And in this there is no word whatever of the "Voice." Socrates then acknowledged himself to be ever acting under the Divine will, which, when impulsive, he calls ο θεος; when restraining, το, or τι δαιμονιον σημειον. All men thus acting who obey the Divine will, this influence was only so far *peculiar* to him as he was ever recognising it, making it a part of his confessed creed; and, from this constant attention to it, becoming ever more conscious of it.

Though this seems to have been perfectly understood by his friends, yet from various causes a different impression arose subsequently. For purposes of their own, his accusers interpreted this mode of speaking into an attempt to introduce strange gods into Athens, and to throw discredit upon the ancient deities. His friends again, and his admirers in after times, personified this Voice, by way of magnifying, as they supposed, the importance of their master, as having been under an especial supernatural influence. And lastly, other writers have brought it forward as a proof that the pagan philosophers had commerce with evil spirits. Thus, Tertullian, in his "Apology," says that "Socrates undertook nothing without the privy counsel of his demon; and no wonder, when this familiar is said to have kept him close company from his childhood to the conclusion of his life; continually, no doubt, injecting dissuasives from virtue."

"That which Plutarch and other admirers of Socrates conceived as a Demon or intermediate Being between gods and men, was looked upon by the fathers of the Christian church as a devil; by Le Clerc as one of the fallen angels; by some other modern commentators as mere ironical phraseology on the part of Socrates himself." *

3. That the reveries of Socrates were of the nature of ecstasy or trance, is unsupported by any evidence; there is, however, some to the contrary. For having fallen into one of them on his way to the "Banquet" with Aristodemus, he withdrew into a porch, and stood still, as in contemplation; and a servant having been sent out to summon him, he *refused* to come in. All this bears no similarity to the insensibility of trance. As before remarked, they were probably instances of profound meditation.

4. His belief in dreams can scarcely be gravely brought forward as even a collateral proof of unsoundness of mind. This was the age when oracles, omens, and dreams were counted amongst the most important guides in all matters; and if on one or two occasions Socrates showed that he was not entirely free from the belief of his country, it can scarcely be considered a ground for reproach. Much more surprising would it have been had not some tincture of superstition adhered in those days, even to so original and gigantic a mind as his.

5. Our limits do not permit us to examine in detail the alleged instances of prophetic power which he claimed. On some few occasions he did predicate what the result would be, as to good or evil, of certain both personal and political acts. But he generally gave the reasons for these conclusions, as has been before remarked concerning the restraining power of the Demon, derived from ordinary rational laws. On this point Mr. Madden observes, in his recent able and deeply interesting work:†

"It may be presumed that the demon of Socrates was nothing more than the rectitude and force of his judgment, which, acting according to the rules of prudence, and with the aid of long experience, supported by wise reflections, made him foresee the events of those things, with regard to which he was either consulted by others, or deliberated upon himself."

6. Socrates was undoubtedly a very eccentric man, but eccentricity is not insanity. He was certainly guilty of having a hole in his coat; he went about barefoot; if he had no supper, he would sometimes prefer to go without rather than to ask for one. A very tiresome one, too; for, like a gad-fly (to use his own expression), he would fix himself upon some puffed-up sophist, and with his endless—"Tell me, now"—"But explain to me"—he would drive the unfortunate wight into such a maze of contradictions as to expose his profound ignorance always to the bystanders, and sometimes to himself. He could not

* Grote's "History of Greece," vol. viii. p. 560.

† "Phantasmata." By R. R. Madden, F.R.C.S. 1857.

forget this even when before his judges. If Melitus could feel at all, he must have wished himself rather in the place of the accused than the accuser. But in all this there is no sign of madness; perhaps this must be sought in his moral eccentricities—for he was temperate in a circle where the drunken Alcibiades was held to be the type of all that was excellent in man; he was pure where impurity assumed its most disgusting aspects; he was virtuous and upright where selfishness was the only recognised law; he was modest where bloated self-conceit and intellectual pride were rampant; above all, he was poor when he might have had boundless wealth.

As to his positive and direct claims to be considered a man of sound mind, these are sufficiently illustrated by the themes of his perpetual teaching—a teaching that only ended with his life. These were—modesty, self-distrust, the necessity for learning, love of parents, temperance, chastity, obedience to the laws, piety towards the gods, faith in their providence, and the recognition of their benefits; a firm belief in the immortality of the soul, and the certainty of a future state of rewards and punishments, according to the deeds done on earth.

In reference to the latter themes, one passage from the "Phædo" merits quotation, as indicating strongly the very far advance which he had made in penetrating things truly divine. Having enumerated certain vices, he adds:—

> "True virtue is a purification from such things; and temperance, justice, fortitude, and wisdom itself, are a kind of initiatory purification. And those who instituted the mysteries for us appear to have been by no means contemptible, but in reality to have intimated long since that whoever shall arrive in Hades unexpiated and uninitiated, shall lie in mud; but he that arrives there purified and initiated, shall dwell with the gods."

We may not dwell further upon the character and teaching of this wonderful man. We protest against the endeavours to demonstrate a morbid alienation in the mind of one to whom, of all others, philosophy is most indebted; and conclude with Mr. Grote, that "no man ever looked upon life with a more positive and practical eye; no man ever pursued his mark with a clearer perception of the road which he was travelling; no man ever combined in like manner the absorbing enthusiasm of the missionary, with the acuteness, the originality, the inventive resource, and the generalizing comprehension of a philosopher."

